# Correction: Boosting or inhibiting - how semantic-pragmatic and syntactic cues affect prosodic prominence relations in German

**DOI:** 10.1371/journal.pone.0336431

**Published:** 2025-11-07

**Authors:** The PLOS One Staff

The correct formatting of examples four and five is:

(4) I went to the dentist yesterday. (5) John got a new car,

I’d like to STRANgle the butcher. [48] and also BILL bought himself a car.

r-given r-new

l-new l-given

In Reading material subsection of Materials and Methods, the passages after the first paragraph should be italicized:

*Maria hat im Büro eine Kollegin, mit der sie sehr gut befreundet ist. Fast jeden Tag hat sie ein kleines Geschenk für Maria dabei, das sie ihr in der Mittagspause überreicht. Heute hat die Kollegin* ***eine Praline (Target1)*** *mitgebracht. Maria war noch am Arbeiten, als die Kollegin zu ihr an den Schreibtisch kam. Dann hat die Kollegin* ***die Praline (Target2)*** *ausgepackt. Sie war von einer edlen Marke und Maria hat sich sehr gefreut.* ***Die Praline (Target3)*** *war mit Karamell und Nüssen gefüllt. Als Kind war sie mit Süßigkeiten sehr wählerisch, was auch mit einem Erlebnis auf ihrem achten Geburtstag zusammenhängt. Damals hat Maria* ***eine Praline (Target4)*** *gegessen. Sie hat aber nicht gewusst, dass die manchmal mit Alkohol gefüllt sind. Maria hat sich geekelt und danach sehr lange gar keine Schokolade mehr angerührt. Diese Zeiten sind zum Glück vorbei und heute liebt Maria Schokolade aller Art.* ***Die Praline (Target5)*** *wird sie gleich als Vorspeise essen.*

*Maria has a colleague in the office with whom she is very good friends. Almost every day she brings a small gift for Maria, which she presents to her during the lunch break. Today, the colleague brought* ***a chocolate candy (Target1)****. Maria was still working when the colleague came to her desk. After that, the colleague unpacked* ***the chocolate candy (Target2)****. It was from a noble brand and Maria was very happy.* ***The chocolate candy (Target3)*** *was filled with caramel and nuts. As a child, she was very picky with sweets, which is related to an experience on her eighth birthday. At that time Maria ate* ***a chocolate candy (Target4)****. But she didn’t know that sometimes they are filled with alcohol. Maria was disgusted and didn’t touch any chocolate for a very long time afterwards. Fortunately, these times are over and today Maria loves chocolate of all kinds.* ***The chocolate candy (Target5)*** *she will eat right away as an appetizer.*

Fig 10 is uploaded incorrectly. Please see the correct Fig 10 here.

**Fig 10:**
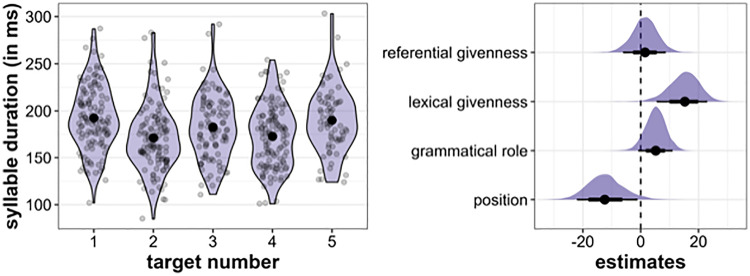
Results for syllable duration. Left: Distribution of syllable duration (in ms) across the five target word positions. Right: Posterior estimates for the effects of referential givenness, lexical givenness, grammatical role and position on syllable duration as predicted by the model, means, 66% (thick horizontal lines) and 90% credible intervals (thin lines).

The publisher apologizes for the errors.
