# Supplementary material for: Boosting or inhibiting - how semantic-pragmatic and syntactic cues affect prosodic prominence relations in German
Source: PLoS One. 2024 Apr 18;19(4):e0299746. doi: 10.1371/journal.pone.0299746 (PMC11025757; doi:10.1371/journal.pone.0299746)
Supplement: Attachment — Submitted filename: Response to Reviewers_Baumann-Lorenzen.docx [file pone.0299746.s001.docx]

Köln, 22 January 2024

Dear Dr. Blake,

We are happy to submit our revision for the manuscript entitled “Boosting or inhibiting - How semantic-pragmatic and syntactic cues affect prosodic prominence relations in German”, authored by Stefan Baumann (corresponding author), and Janne Lorenzen.

We thank you and the reviewer for the very helpful feedback. We have considered the points raised and give a point-by-point description below (our answers are given in italics).

Furthermore, please change our online submission form on our behalf by adding to the statement on financial disclosure ("This work was supported by the German Research Foundation (DFG) as part of the SFB1252 Prominence in Language (Project-ID 281511265), project A07 Metrical prominence – Scales and structures.") the following sentence: "The funders had no role in study design, data collection and analysis, decision to publish, or preparation of the manuscript."

Finally, we included our full ethics statement in the "Participants and experimental procedure" section of our manuscript file, including the full name of the ethics committee who approved our study.

With kind regards,

Stefan Baumann and Janne Lorenzen

**Response to Reviewer 1**

Introduction:
The Introduction section is well written and the research question is described clearly. The previous literature is reviewed such that a clear picture of the research question emerges.

Section 2.2:
It is mentioned that participants "originat[e] from five different federal states of Germany." Are there any potential differences between dialects of the participants? If not, state this explicitly.

*Thank you for this question. We added the statement "None of them spoke in a non-standard variety" to clarify this point.*

Is there any possible effect of gender? With only 4 male and 11 female speakers, the data is not balanced across gender. If there are differences, it would not necessarily be clear then due to this imbalance. This should be mentioned explicitly as a limitation.

*We added this information to a paragraph in the Discussion section, when talking about speaking style and focus. In particular, the following sentence was included: "Gender did not play a role, although we have to state that the dataset is not balanced across gender (see section Participants and experimental procedure)."*

Section 2.3:
Include the inter-rater reliability between the two annotators for word prominence level annotation. Also, how many words required a consensus from a third expert?

*We included the inter-rater agreement for all prosodic annotations (phrase boundary, DIMA tone level, GToBI accent types and perceived prominence). The inverse of the agreement rates constitute the amount of annotations requiring a consensus with the third expert (e.g. an agreement rate of 94% means that we had to decide on a consensus annotation in 6% of the cases).*

When normalizing F0 by gender, what was the justification to use 75 Hz for males and 120 Hz for females? Why not normalize by individual mean F0 (per participant) instead to account for participant differences (regardless of gender)?

*We agree that a participant-specific normalization is the more thorough process and have adjusted the TCoG scaling variable and its analysis in this regard. The results do not change following this procedure.*

Section 2.4: The statistical analysis is nicely presented.

*Thank you. :-)*

Section 4: The authors’ discussion following example (9) offers a plausible answer to the question of why initial position tends to attract nuclear accent: It’s a global preference and doesn’t depend on anything else. The authors might consider reframing their initial predictions then, or at least entertaining this possibility from the outset in the Introduction section rather than introducing it for the first time in the Discussion section. It need not be presented as such a surprising result, in other words.

*First of all, initial position generally attracts accents, but mainly prenuclear ones (not nuclear accents).*

*Secondly, we still want to say that we did not predict the initial position to be that strong, as we want to be transparent about our initial assumptions, but we tried to attenuate the "surprisal" to some extent, as suggested, by changing the wording, e.g. to "contrary to our predictions" (Conclusions section). We also prepared the rhythmical frame idea in the Introduction a bit more. In the Abstract, we deleted "somewhat surprisingly" completely.*

*Thank you again for your valuable feedback!*
